# Somatic Profile of Competitive Sport Climbers

**DOI:** 10.2478/v10078-011-0044-7

**Published:** 2011-10-04

**Authors:** Paweł Tomaszewski, Jan Gajewski, Joanna Lewandowska

**Affiliations:** 1 Department of Statistics and Computer Sciences, Jozef Pilsudski University of Physical Education, Warsaw, Poland; 2 Department of Biomechanics, Institute of Sport, Warsaw, Poland; 3 Department of Anthropology, Jozef Pilsudski University of Physical Education, Warsaw, Poland

**Keywords:** anthropometry, climbing, sport performance

## Abstract

Since rock climbing grows in popularity, the number of the respective scientific reports increases. However, those concerning anthropometric profile of elite climbers are scarce and inconsistent, thus the aim of the study was to describe the anthropometric characteristics of competitive sport climbers. Male rock climbers (n = 21) aged 17 – 29 years took part in the study; their climbing ability ranged from 6b to 8c in the French scale. Body height, body mass, arm span, length and girths of both extremities, shoulder and pelvis widths, as well as thickness of 5 skinfolds were determined. From these, body mass index (BMI), body fat content and selected anthropometric indices were calculated. Data collected for climbers were compared with those of untrained students (n = 165) of Warsaw Technical University. Although no between-group differences were found for body height, body mass, BMI or body fat content, the climbers exhibited significantly (p<0.001) lower pelvis-to-shoulder ratio, longer lower extremities (p<0.05), and greater arm length and arm span (p<0.001) compared to untrained students. The results of this study do not support the view that climbers are small in stature and of low body mass. It seems that the core of the issue is not in body size but rather in specific body proportions and this may be of great importance in selecting subjects to competitive sport climbing.

## Introduction

Rock climbing was recognized for many years as a recreational activity or entertainment that links in its peculiar way the sport with the beauty of nature. As a form of spending leisure time, the definition of climbing is located between the amateur or professional sport, recreation and qualified tourism. However, direct rivalry and the standards unifying conditions of climbing competition refer to the modern definition of a professional sport (Lewis and Cauthorn, 2000). Irrespectively of the definition assumed, there is no question that in recent years rock climbing has become increasingly popular and the standard of climbing continues to rise. It has become a competitive international sport with an annual international World Cup competition circuit beginning in 1989 on artificial climbing walls. Currently, there are several types of climbing competitions, such as lead climbing, speed climbing, bouldering and ice climbing, which have led to the progress.

A sport-specific somatic build is believed to be one of the determinants of top performance in many sports and an athlete’s anthropometric characteristics can play a major role in determining sport success (Reilly et al., 1990). Moreover, specific somatic predispositions are frequently considered one of the key elements in the process of sport selection and talent identification (Aitken and Jenkins, 1998; Gil et al., 2007a; 2007b). While it is obvious that in some sports skill and physical fitness may also be the key contributing factors, this has not stopped anthropometric profiling being used in a number of sports, e.g. rowing (Bourgois et al., 2000), kayaking (Ackland et al., 2003), cycling (Brian et al., 1989) or in team games (Chaouachi et al., 2009; Duncan et al., 2006; Duthie et al., 2006; Reilly et al., 2000). Several attempts at describing anthropometric and physiological characteristics of climbers were also made (Giles et al., 2006; Grant et al., 1996; Mermier et al., 2000; Sheel, 2004; Watts et al., 2003). In those studies, elite climbers were characterized in general as small in stature, with low body mass and body fat content and high handgrip strength related to body mass. Despite an extensive research in that area, there is still some debate and conflicting evidence in the climbing literature as to which physiological and anthropometric factors are important in determining climbing performance. In activities like climbing, where body mass is repeatedly lifted against gravity, extra mass, in the form of fat or large muscle mass is regarded disadvantageous (Grant et al., 1996). A lighter mass reduces force output by muscles that would be required to sustain position and maintain given hand configuration. This could result in a slower rate of fatigue in smaller climbers compared with their heavier counterparts. Average body height, and especially large arm span, enable climbers to ascend the route more effectively and may minimize the required work output when moving along a climbing route. Such physical attributes would prove advantageous as the absolute workload and force used to support and move the body would be reduced. It may be thus expected that low body mass/fat, relatively long upper extremities and high grip strength may be beneficial for achieving top performance in climbing and would characterize elite climbers. The aim of the study was thus to determine the anthropometric profile of competitive sport climbers.

## Material and Methods

21 male climbers volunteered to participate in the study conducted during friendly lead climbing competition of a local rank, held in Warsaw in 2007. Their age ranged from 17 to 29 years (mean: 22.4 years) and self-reported climbing ability defined according to the most difficult route ever made from 6b to 8c in French scale. Each climber had a recognized training experience exceeding 3 years. All climbers gave their written consents to participate in the study which was approved by the local Ethics Committee.

All anthropometry measurements were made before competition in resting state according to established procedures (National Center for Health Statistics, 2004). Body height and mass were assessed in standing position, barefooted, to the nearest 0.1 cm and 0.1 kg, respectively; body height was additionally recorded in sitting position (“sitting height”). Arm span was measured in standing position, arms abducted horizontally, the greatest tip-to-tip distance between the extended fingers being recorded. Arm length was measured as the distance between the acromion and dactylion, leg length being presumed equivalent to the difference between standing and sitting heights. Arm circumference was measured at muscle contraction on biceps brachii muscle bulk using measuring tape; forearm and calf circumferences were measured around the maximum girth of the respective part of the limb. Shoulder and pelvis widths were measured as the distance between the most lateral points on the acromion processes or iliac tubercles, respectively. Elbow and knee widths were measured with the caliper as the distance between the lateral and medial humeral or femoral epicondyles, respectively. Skinfold thickness was measured to the nearest 0.5 mm with a calibrated caliper at five sites: abdomen, biceps, suprailiac, subscapula and calf. All measurements were taken on the participant’s right side.

From collected data, the following indices were calculated:

– BMI,
– Rohrer’s index - the ratio of body mass (in grams) to body height^3^ (in cm) × 100,
– Body fat content (%FAT) – estimated using the Keys-Brozek equation (Brozek, 1966) for body density assessed from logarithmic equation for abdominal and biceps skinfold thickness (Piechaczek, 1975).
– Manouvrier’s index – the ratio of leg length to sitting height,
– Arm length index – the ratio of arm length to body height,
– Arm span index (ape index) - the ratio of arm span to body height,
– Upper extremity girth index - the ratio of forearm to arm circumference,
– Pelvis-to-shoulder ratio

Additionally, somatotype components were determined for every participants according to the Heath-Carter method (Carter, 1996).

Data collected for climbers were standardized against means and standard deviations obtained for untrained, sedentary students (n = 165) of Warsaw Technical University (Piechaczek et al., 1996). Student’s t-test for independent data was used to assess the between-group differences. Pearson’s correlation was used to assess relationships between the studied variables; partial correlation coefficients were calculated to reveal possible contributions of somatic variables to the climbing ability. The significance level for all estimates was set at α=0.05.

## Results

**Figure 1:**
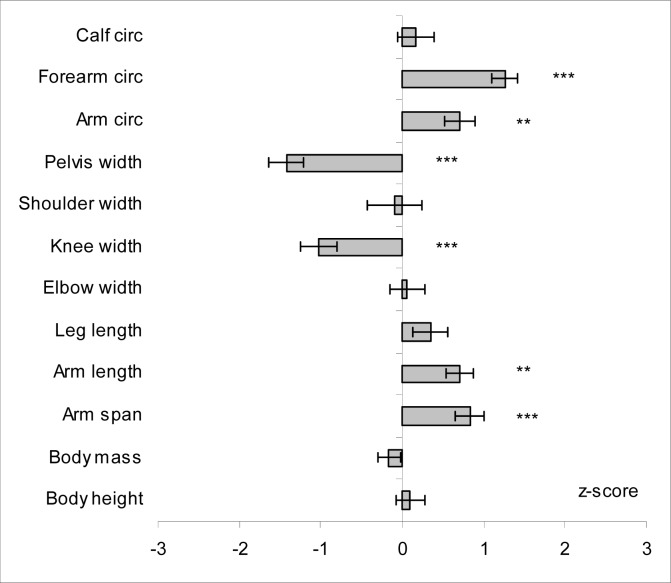
Mean values (±SE) of anthropometric variables recorded in climbers (n = 21) standardized against means and standard deviations obtained for untrained students (Piechaczek, 1996) Legend: Circ - circumference; Significantly different from students: **p<0.01; ***p<0.001

**Figure 2:**
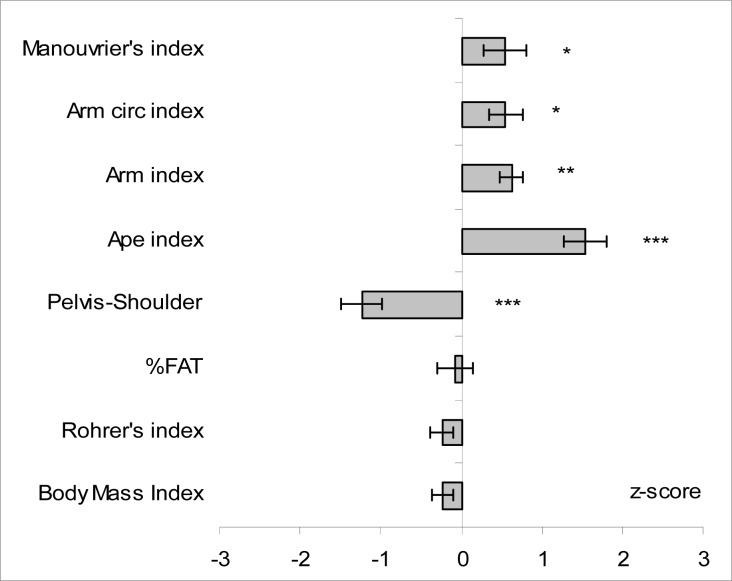
Mean values (±SE) of somatic indices determined in climbers (n = 21) standardized against means and standard deviations obtained for untrained students (Piechaczek, 1996) Legend: Circ- circumference, %FAT – Body fat content; Significantly different from students: *p<0.05; **p<0.01; ***p<0.001

Mean standardized values of anthropometric variables and somatic indices are presented in Figures 1 and 2, respectively.

Body height and body mass of studied subjects were rather average as no significant between group differences were noted for those variables – mean body height was 180.0 ± 4.95 and 179.4 ± 6.19 cm and mean body mass was 70.7 ± 5.93 and 72.1 ± 8.96 kg for sport climbers and untrained students respectively. No significant between-group differences were noted for BMI and Rohrer’s index (RI); all climbers had normal body mass (18.50 > BMI > 24.99) according to WHO classification (WHO, 2004). Likewise, in case of weight/height indices, mean body fat percentage recorded in climbers was comparable to this observed in untrained students and amounted to 15.4%. However, when classified by Heath-Carter somatotype components, endomorphy component that reflects adiposity had the lowest contribution in climbers’ somatotype; the mean value being significantly (p<0.001) lower than that observed in untrained students (2.4 ± 0.79 vs. 3.6 ± 1.48, respectively). Regardless of comparable body height, climbers had significantly greater arm span and arm length (by about 6 and 2.5 cm, respectively) what was reflected in ape index and arm length index, the respective values being by about 1.5 (p<0.001) and 0.6 SD (p<0.01) greater than observed in untrained students, respectively. Additionally, climbers exhibited significantly greater values in arm (32.7 ± 2.09 vs. 30.9 ± 2.52 cm) and forearm circumferences (28.3 ± 1.28 vs. 26.02 ± 1.80 cm) and in upper extremity girth index, while no differences were found for elbow width. On the other hand, climbers had by 1 SD (p<0.001) lesser knee width while no between-group differences were found for calf circumference. Moreover, climbers exhibited by about 1 SD less in pelvis-to-shoulder ratio comparing to untrained students. Likewise, for upper extremities climbers had significantly (p<0.05) longer lower limbs as expressed by the Manouvrier’s index.

In order to reveal possible relationships between somatic indices and subjects’ climbing ability, Pearson’s correlation coefficients and partial correlations were calculated. Apart from the obvious relations between the body fat and weight-to-height indices or between indices pertaining to the length of upper limb, significant negative correlations were found only for %FAT and ape index (−0.594; p<0,01) and for arm circumference index and BMI (r = −0.497; p<0.05) or RI (r = −0.587; p<0.01). Self-reported climbing ability significantly correlated with %FAT (r = −0.614; p<0.01); besides that, no significant correlations with somatic indices were noted and none of the partial correlations proved significant. Only the ape index tended to correlate with the self-reported climbing ability (r = 0.397; p = 0.083).

## Discussion

Despite the growing number of reports on rock climbing, those concerning anthropometric characteristics of climbers are rather scarce and inconsistent. The results of this study do not support the view of Watts et al. (2003) that climbers are small in stature with low body mass as no differences between the climbers and untrained controls were found for basic somatic features and body size-related indices. Body height and body mass of climbers were rather average and amounted to 180.0 cm and 70.7 kg, respectively, what was in line with the observations of Billat et al. (1995) and Grant et al. (1996) who found rock climbers to be on average 180 cm in height and weighing slightly above 71 kg. Moreover, all studied climbers had normal BMI (between 18.50 and 24.99) and body fat percentage (15%) almost equivalent to the values observed in untrained subjects. Although Watts et al. (2003) found no significant differences between climbers and controls for absolute BMI scores or for BMI expressed as a percentile score, they assessed body fat percentage in elite climbers being as low as 5% by using sum of skinfolds and the Jackson and Pollock’s method. This extremely low value found no confirmation in this study and in reports of other authors in which the estimated body fat content was 14 and 15.3% in elite and recreational climbers, respectively (Grant et al., 1996; 2001), and 5 – 15% in male athletes of other sports (Corbin et al., 2000). Such variations in data, resulting in mixed conclusions, may be attributable to the methods of assessment used. The Jackson and Pollock’s method of calculating body composition using three sites of assessment was used in the study of Mermier et al. (2000) while the Durnin and Womersley’s method using four sites of assessment was employed by Grant et al. (1996). This makes it increasingly difficult to make direct comparisons between studies and becomes a limitation when attempting to draw conclusions. All these limitations incline some authors to conclude that there is little evidence to support the view that low body fat percentage contributes to successful climbing (Grant et al., 1996; 2001) while others consider it along with other trainable variables particularly important to climbing performance (Watts et al., 1993; Mermier et al., 2000). In this study, significant correlation was found between %FAT and self-reported climbing ability (r = −0.614; p<0.01) what partly confirms the importance of low body fat percentage in climbing performance. Such differences within the literature and the results obtained in this study would suggest that low body mass and body fat percentage are not a prerequisite for elite-level climbing, although they may be seen as beneficial.

A long reach relative to height is thought to improve climbing performance (Watts et al., 2003). The climbers in this study had significantly (p<0,001) greater arm length and arm span and significantly higher ape index scores than the controls (1.05 vs. 1.02, respectively), the ape index values being similar to those observed by Mermier et al. (2000) for adult male climbers (1.0). Moreover, ape index nearly significantly correlated with climbing ability (r = 0.397; p = 0.083) and the low correlation coefficient was probably due to the relatively small variability in ape index among climbers (SD = 0.02). Similar results were obtained by Watts et al. (2003) who found no significant correlation between ape index and climbing ability, observing the same variation in ape index. The authors concluded that ape index could be more important when other traits were equivalent.

It might be hypothesized that a lower biiliocristal/biacromial ratio that indicates a more triangular torso would be advantageous in climbing and, due to its specificity, would be thus more pronounced in elite climbers. This hypothesis was confirmed in this study – the climbers exhibited significantly (p<0,001) lower pelvis-to-shoulder ratio compared to untrained students. However, presented results are not in line with those of other authors (Watts et al., 2003) who found a narrower biacromial breadth (28.1 ± 2.5 vs. 35.7 ± 4.1 mm) relative to biiliocristal breadth in climbers than in controls (24.1 ± 2.6 vs 26.2 ± 2.6 mm, respectively). It is thus difficult to agree with the proposed explanation of authors that narrower shoulder structure in climbers can be advantageous as it could, to some extent, account for the observed lower body mass. It rather seems that greater biacromial breadth combined with the relatively large ape index could have affected the reach distance in given body position thus having a positive impact on climbing performance. Moreover, sport climbers had significantly (p<0.001) higher values of upper extremity girth index (84.5 ± 4.27 and 86.8 ± 4.08, respectively) and significantly (p<0.05) longer lower extremities (by Manouvrier’s index). All these results may be indicative of some specific anthropometric profile of competitive climbers; it is, however, difficult to discuss those aspects since the results pertaining to dimensions of lower limbs were virtually neglected in the literature.

Unexpectedly, no significant relationships were found in this study between either somatic variables or self-reported climbing ability and most of the somatic indices studied. That result was partly confirmed by the observations of Watts et al. (1993) who entered trainable variables, along with other anthropometric and physiological variables, into a multiple regression model and found that only %FAT and relative grip strength were considered significant predictors of climbing ability. Thus, it was assumed that trainable variables are most important to climbing performance in elite climbers and thereby questioned that a sport climber must have specific anthropometric characteristics to be successful.

When commenting the results of this study, the quite large scatter of climbing skills, ranging from 6b to 8c, may be regarded as certain limitation and precise conclusions regarding the somatic profile of elite climbers may prove difficult. However, since achieving the 6b level requires at least several months of regular training, it may be supposed that subjects who were not physically/somatically (or mentally) predisposed to climbing could have dropped out. It seems thus justified to consider the studied climbers as to some extent homogenous somatically and to look for somatic characteristics even in such a diversified group. Moreover, clear and convincing, highly significant differences between climbers and untrained subjects presented here give an outlook on somatic characteristics of competitive climbers.

In general, it appears that success in climbing is not only related to individual anthropometric or physiological variables but is the result of a complex interaction of physiological and psycho-emotional factors. Summing up, although there is a tendency among elite climbers to share certain anthropometric characteristics, they are not necessarily required to attain the top level of climbing performance. However, low body mass, body fat percentage and average body height, and a higher score of ape index, may be considered beneficial for sporting success in climbing. Because no between-group differences were noted for basic somatic features and body-size-related indices, it may be hypothesized that the core of the issue is not in general body size but rather in specific body proportions and that may be of great importance in selecting subjects to professional participation in sport climbing. Hence, at the early stage of selection, subjects with relatively long extremities and narrow hips can be regarded as having chances for practicing rock climbing and when properly trained could achieve top level of sport performance.
